# Symptom burden & quality of life among patients receiving second-line treatment of metastatic colorectal cancer

**DOI:** 10.1186/1756-0500-5-314

**Published:** 2012-06-20

**Authors:** Mark S Walker, Elaine Yu Pharm, Jiandong Kerr, Yeun Mi Yim, Edward J Stepanski, Lee S Schwartzberg

**Affiliations:** 1 ACORN Research, LLC, Memphis, TN, USA; 2 Genentech, South San Francisco, CA, USA; 3 The West Clinic, Memphis, TN, USA; 4 6555 Quince, Suite 400, Memphis, TN, 38119, USA

**Keywords:** Bevacizumab, Cetuximab, Chemotherapy, Health outcomes, Dermatologic symptoms

## Abstract

**Background:**

Bevacizumab (B) and cetuximab (C) are both approved for use in the treatment of metastatic colorectal cancer (mCRC) in the second-line. We examined patient reported symptom burden during second-line treatment of mCRC.

**Methods:**

Adult mCRC patients treated in the second-line setting with a regimen that included B, C, or chemotherapy only (O) and who had completed ≥ 1 Patient Care Monitor (PCM) surveys as part of routine clinical care were drawn from the ACORN Data Warehouse. Primary endpoints were rash, dry skin, itching, nail changes, nausea, vomiting, fatigue, burning in hands/feet, and diarrhea. Linear mixed models examined change in PCM scores across B, C and O (B = reference).

**Results:**

182 patients were enrolled (B: n = 106, C: n = 38, O: n = 38). Patients were 51% female, 67% Caucasian, with mean age of 62.0 (SD = 12.6). Groups did not differ on demographic or clinical characteristics. The most common second-line regimens were FOLFIRI ± B or C (23.1%) and FOLFOX ± B or C (22.5%). Results showed baseline scores to be strongly predictive of second-line symptoms across all PCM items (all p’s < .0001 except for Rash, p = .0013). Controlling for baseline, patients on B tended to have more stable and less severe symptoms. Patients on C had more severe rash, dry skin, and itching and had nail change scores that worsened faster than did B patients.

**Conclusions:**

Patients receiving second-line treatment for mCRC with B report less symptom burden, especially dermatologic, compared to patients treated with C.

## Background

The American Cancer Society estimates that approximately 141,210 people will be diagnosed with colorectal cancer (CRC) in the United States in 2011 with roughly 49,380 people dying of the disease during the same time frame [1]. CRC is the third most commonly diagnosed cancer among both men and women and the third leading cause of cancer death overall. Incidence and death rates for CRC increase with age with 90% of new cases and 94% of deaths occurring in individuals 50 years of age and older [1].

CRC is a cancer that starts in the large intestine or the rectum. Cancer cells eventually spread to nearby lymph nodes and subsequently to more remote lymph nodes and other organs in the body with the liver and lungs being the most common metastatic sites. Approximately 30% of all patients with CRC have metastatic disease at diagnosis, and between 40% and 65% of all patients diagnosed with CRC will eventually develop metastatic or advanced disease [2,3].

The management of patients with metastatic colorectal cancer (mCRC) has changed dramatically over the last decade. Historically, 5-fluorouracil (5-FU) was the only active agent in CRC. The introduction of several new chemotherapeutic (irinotecan, oxaliplatin) and biologic agents (cetuximab, bevacizumab, panitumumab) into clinical practice have resulted in significant gains in response rates and overall survival [4-6].

The therapies recommended by the National Comprehensive Cancer Network (NCCN) after the first progression in patients who have received prior 5-FU/leucovorin (LV) based or capecitabine based therapies are dependent on the initial treatment regimen [7,8]. If FOLFOX or CapeOx based therapies are used as first-line, FOLFIRI, with or without cetuximab or panitumumab (KRAS wild type tumor only), and irinotecan in combination with cetuximab (KRAS wild type tumor only) or as a single agent is recommended. In patients who received a FOLFIRI based regimen as the first-line therapy, FOLFOX or CapeOx, cetuximab plus irinotecan, or single agent cetuximab or panitumumab (for those not appropriate for the combination with irinotecan) are recommended options. For patients who received 5-FU/LV or capecitabine without oxaliplatin or irinotecan as initial therapy, options after first progression include FOLFOX, CapeOx, FOLFIRI, single agent irinotecan, or irinotecan plus oxaliplatin. For patients who received FOLFOXIRI as initial therapy, cetuximab plus irinotecan or cetuximab or panitumumab alone are recommended options for those with wild-type KRAS gene. NCCN guidelines also note that bevacizumab, if not used in initial therapy, may be appropriate to add to chemotherapy following progression of metastatic disease.

Treatments for mCRC are mainly palliative. They seek to increase the duration, and maintain or improve the quality of the patient’s remaining life, a difficult task given the toxicity of the given chemotherapy combinations [5]. The addition of bevacizumab or cetuximab to these regimens may result in somewhat different toxicity profiles. Package inserts for both products report that common reactions include headache and diarrhea [9,10]. Bevacizumab labeling also reports epistaxis (nosebleed), hypertension, rhinitis, proteinuria, taste alteration, dry skin, rectal hemorrhage, lacrimation disorder, back pain and exfoliative dermatitis to occur in ≥ 10% of cases [10]. Cetuximab labeling reports rash, pruritus, nail changes and infection to occur in ≥ 25% of cases [9].

Research has suggested health-related quality of life (HRQoL) is positively associated with subsequent survival duration [11-13], making it an important consideration in decisions related to treatment of patients with metastatic disease. The total number of side effects and the intensity of specific toxicities, most notably fatigue, insomnia, nausea, vomiting and diarrhea, have been shown to markedly affect patient satisfaction with HRQoL [14-17]. The specific effect of other toxicities, such as dermatologic toxicities, on patient satisfaction with HRQoL is less well understood although they are known to occur with frequency in some treatments [18,19].

Although many studies have examined HRQoL among patients receiving first-line treatment of mCRC [20-22], few have examined symptom burden and HRQoL in the second-line. In the few that have, all have been conducted in the clinical trial setting. In addition, few studies have conducted multi-regimen comparisons involving recommended therapies. The goal of the current study was to describe symptom burden among patients treated for mCRC in community settings who received second-line regimens that contained (1) bevacizumab, (2) cetuximab or (3) chemotherapy regimens without the addition of a monoclonal antibody (designated Chemotherapy Only).

## Materials and methods

### Patients & setting

Potentially eligible patients were identified through review of electronic records of patients at community oncology practices represented in the ACORN Data Warehouse. The ACORN Data Warehouse contains electronic medical record, billing, and patient reported outcome data from 13 community oncology practices. Data are extracted electronically, refreshed weekly, and used as source data for cancer research. Medical charts were subsequently reviewed to determine final study eligibility. The final study sample was drawn from patients in seven affiliated practices. All study procedures were approved by IntegReview, a commercial IRB in Austin, TX.

Patients were eligible if they were at least 18 years of age, had a confirmed diagnosis of stage IV colorectal cancer, had experienced at least one disease progression after diagnosis with mCRC, and received second-line treatment with a qualifying regimen. Qualifying regimens were those that included bevacizumab, cetuximab, or chemotherapy alone without monoclonal antibodies. We considered regimens that included panitumumab, but the sample of patients who received second-line panitumumab was too small for meaningful comparison. Eligibility also required that patients had completed at least one Patient Care Monitor (PCM) assessment, described further below, during the period in which they were receiving second-line treatment for mCRC.

### Primary study endpoint

The primary study endpoint was patient reported outcomes as indicated by the PCM assessment. This included individual PCM items such as ‘rash,’ ‘dry skin,’ ‘Itching,’ ‘nail changes,’ ‘nausea’, ‘vomiting,’ ‘diarrhea,’ and ‘burning sensation in hands or feet.’ It also included PCM index scores, described below. Although other symptoms were of interest for the study, the range of items under consideration was limited by those available as part of the PCM. The focus of reporting in this paper is the individual PCM items that describe specific patient symptoms, listed above.

PCM, version 2.0, is an 86-item self-report measure that assesses physical symptoms, psychological symptoms and physical functioning, and asks patients to rate the severity of symptoms on an 11 point (0 to 10) Likert-type scale, where higher scores reflect more severe symptoms. The PCM is administered via touch screen tablet personal computer as a routine part of care at participating community oncology practices. In addition to scores on individual PCM items, the PCM produces standardized index scores (T scores) for six screening scales in which higher scores denote more severe symptoms. The indices are: General Physical Symptoms, Treatment Side Effects, Despair and Depression, Acute Distress, Impaired Ambulation, and Impaired Performance. The PCM has been shown to be valid for assessing symptom burden and HRQoL in cancer patients and has been used in a number of studies [23-27]. The focus of this study was on symptom burden.

### Other measures

Demographic characteristics included age, gender, ethnicity, body mass index, and geographic location. Clinical characteristics included performance status, primary CRC site, sites of metastases, tumor histology, KRAS status if available, and the presence vs. absence of documented evidence for KRAS testing prior to the start of second-line treatment. Treatment characteristics included first and second-line chemotherapy and/or targeted therapy regimens, including duration of first-line therapy.

### Statistical methods

The underlying populations of patients treated with bevacizumab, cetuximab, or chemotherapy alone from which the sample was drawn were not of equal size; resulting samples in each treatment group were therefore expected to be unequal. In particular, we were aware that more patients with bevacizumab experience were available, and planned for a larger sample in this group to provide increased statistical power for group comparisons.

Descriptive statistics were generated for all demographic, disease, and treatment characteristics. Frequencies and percentages were generated for categorical variables and the mean, standard deviation, median, minimum, and maximum were generated for continuous variables. Chi-square tests of independence and Fisher’s Exact test were used to compare groups across levels of categorical variables. T tests and analysis of variance were used to compare groups on continuous variables.

Linear mixed models were employed to examine change in PCM endpoints over time during the second-line period, across Bevacizumab vs. Cetuximab vs. Chemotherapy Only groups, with the Bevacizumab group defined as the reference for group comparison. Models employed restricted maximum likelihood estimation, with random intercept and random slope for regression of PCM endpoints on interval since starting second-line chemotherapy. Methods generally followed those reported by Cnaan and Little [28,29].

Models included as predictors the interval in days between the start of second-line treatment and the PCM survey (Interval), the treatment group (Group), and the interaction of these two terms. These were included in the models irrespective of statistical significance. Each model also considered the following predictors for inclusion in the model: race group, age at start of second-line therapy, duration in days of first-line therapy, body mass index, whether the patients were receiving chemotherapy or targeted therapy at the time of the PCM survey, first-line treatment regimen as containing oxaliplatin vs. irinotecan vs. neither, and second-line chemotherapy regimen as containing oxaliplatin vs. irinotecan vs. neither.

A second set of models were generated which additionally controlled for baseline PCM values. Baseline values were defined as the PCM score closest in time to the start date of second-line therapy, and within 14 days of the start of second-line therapy. Only patients with a qualifying baseline value were included in this additional analysis. All statistical tests were interpreted at alpha = .05, two tailed, and no adjustment was made for multiple comparisons.

## Results

### Sample development

**Figure 1:**
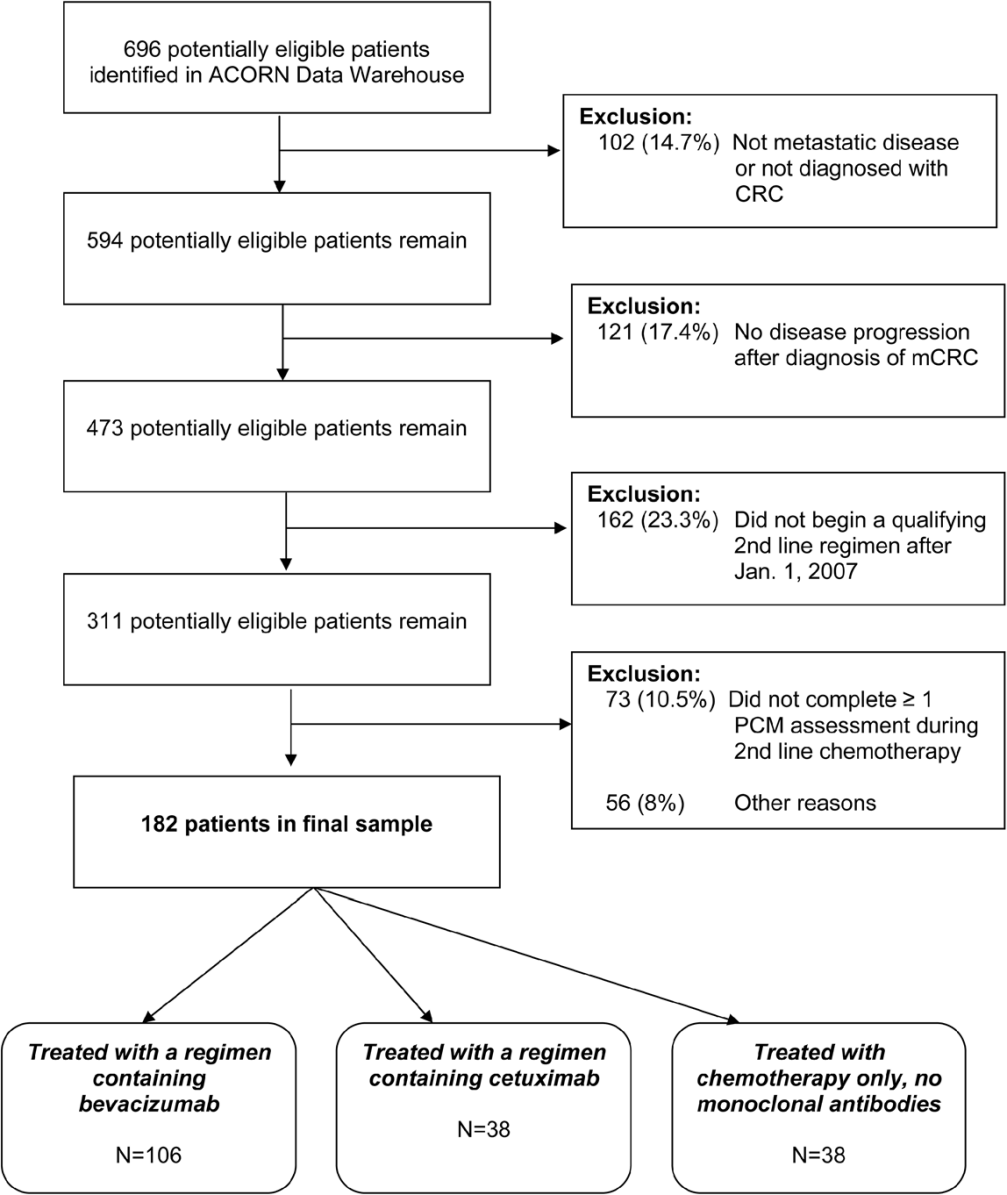
Sample development.

A total of 696 potentially eligible patients were identified, 14.7% of whom on review did not have metastatic disease, or did not have CRC. Primary reasons for exclusion in the remaining potentially eligible subjects included no evidence of disease progression after diagnosis with mCRC (17.4%), initiating second-line therapy prior to January 1, 2007 (23.3%) and insufficient PCM data (10.5%). A total of 182 patients met all eligibility criteria, and represented the final study sample. Figure 1 shows sample development.

### Study population

The sample was largely Caucasian (67.6%) with a mean age of 62.0 (SD = 12.6) years. About half of the sample was female (51.1%). Three quarters of patients resided in the southern United States. By definition, all patients had stage IV disease. The most common primary sites of disease were sigmoid colon, cecum, and ascending colon. More than 90% of patients had adenocarcinoma. The predominant sites of metastasis were liver (65.4%), lung (46.7%), and the peritoneum (23.6%). KRAS test status was unavailable in 55% of patients, primarily due to the fact that 52% of patients initiated second-line therapy prior to June 2008. Among those patients where KRAS testing was known to occur (N = 44), 45.5% had mutant results. There were no significant differences in any of the demographic or clinical characteristics across the three regimen groups.

Fifty eight percent of the sample used a second-line regimen containing bevacizumab, 21% used a second-line regimen containing cetuximab, and 21% used a chemotherapy regimen that did not include a monoclonal antibody. Note, however, that the study sample was a convenience sample—patients were not randomly selected. The distribution of second-line regimens observed in this sample is therefore not assumed to reflect the population distribution of second-line regimens.

The mean duration of first-line therapy across all groups was 212.5 days (SD = 162 days), and this did not significantly vary across second-line regimen group (p = .136). The mean duration of second-line therapy across all groups was 144.7 days (SD = 116.4), but 17 patients had not finished second-line therapy at the time of data collection. Time to event analysis of the duration of second-line therapy was therefore conducted, to provide an unbiased estimate of the duration. This analysis showed the median duration of second-line therapy to be 124 days (Chemotherapy Only: 100 days, Cetuximab: 113 days, Bevacizumab: 136 days). A log rank test comparing duration across second-line treatment groups showed the group differences in duration of second-line therapy to be statistically significant (p = .0399).

Cetuximab patients were more likely than expected to have received an irinotecan-based regimen (*χ*^2^ (186,2) = 8.765, p = .0129). Nearly all patients who received an oxaliplatin-based regimen were in the Bevacizumab group, with about half of Bevacizumab patients receiving oxaliplatin, compared with about 11% in the combined Chemotherapy Only and Cetuximab groups (*χ*^2^ (186,2) = 31.004, p < .001). Bevacizumab patients were also much more likely to have used 5-FU based regimens (*χ*^2^ (186,2) = 21.548, p < .001). There was no significant difference in the rate at which patients received capecitabine as part of their regimen.

Additional detail on the demographic, clinical and treatment characteristics of the population, by treatment group, are reported in Tables 1 and 2.

**Table 1:** Demographic & clinical characteristics by treatment group

**Variables**	**Chemotherapy only N = 38**	**Cetuximab N = 38**	**BevacizumabN = 106**	**Total N = 182**
Gender, n (%)				
Female	23 (60.5%)	14 (36.8%)	56 (52.8%)	93 (51.1%)
Age (years)				
Mean (SD)	60.8 (14.7)	64.6 (11.2)	61.5 (12.3)	62.0 (12.6)
US Region, n (%)				
Northeast	4 (10.5%)	3 (7.9%)	3 (2.8%)	10 (5.5%)
Midwest	0 (0.0%)	2 (5.3%)	4 (3.8%)	6 (3.3%)
South	27 (71.1%)	28 (73.7%)	83 (78.3%)	138 (75.8%)
West	7 (18.4%)	5 (13.2%)	16 (15.1%)	28 (15.4%)
Race, n (%)				
White	26 (68.4%)	26 (68.4%)	71 (67.0%)	123 (67.6%)
Minority	12 (31.6%)	12 (31.6%)	35 (33.0%)	59 (32.4%)
Sub-Total	38 (100%)	38 (100%)	106 (100%)	182 (100%)
BMI				
Mean (SD)	26.3 (6.2)	28.9 (5.2)	26.9 (5.4)	27.2 (5.6)
Disease Stage, n (%)				
Stage IV	38 (100%)	38 (100%)	106 (100%)	182 (100%)
ECOG, n (%)				
0	8 (21.1%)	10 (26.3%)	19 (17.9%)	37 (20.3%)
1	8 (21.1%)	6 (15.8%)	19 (17.9%)	33 (18.1%)
2+	2 (5.3%)	3 (7.9%)	3 (2.8%)	8 (4.4%)
Text indication of impairment	4 (10.5%)	2 (5.3%)	14 (13.2%)	20 (11.0%)
No text indication of impairment	16 (42.1%)	17 (44.7%)	51 (48.1%)	84 (46.2%)
Sites of Distant Metastasis, n (%) *				
Liver	24 (63.2%)	24 (63.2%)	71 (67.0%)	119 (65.4%)
Lung	14 (36.8%)	21 (55.3%)	50 (47.2%)	85 (46.7%)
Peritoneum	9 (23.7%)	6 (15.8%)	28 (26.4%)	43 (23.6%)
Small intestine	0 (0%)	0 (0%)	4 (3.8%)	4 (2.2%)
Other	16 (42.1%)	15 (39.5%)	47 (44.3%)	78 (42.9%)
Not documented	0 (0%)	0 (0%)	1 (0.9%)	1 (0.5%)

* Patients may be represented on more than one row.

**Table 2:** Treatment characteristics by second-line treatment group

	**Chemotherapy only N = 38**	**Cetuximab N = 38**	**Bevacizumab N = 106**	**Total N = 182**
**Regimen used in 1st line therapy, n (%)**				
Oxaliplatin based	5 (13%)	4 (11%)	54 (51%)	63 (35%)
Irinotecan based	21 (55%)	28 (74%)	40 (38%)	89 (49%)
Neither Oxaliplatin nor Irinotecan based	12 (32%)	6 (16%)	12 (11%)	30 (16%)
**Regimen used in 2nd line therapy, n (%)**				
5-FU	0 (0.0%)	0 (0.0%)	1 (0.9%)	1 (0.5%)
5-FU / AMG 706 / Irinotecan / Leucovorin	1 (2.6%)	0 (0.0%)	0 (0.0%)	1 (0.5%)
5-FU / Gamma Interferon / Irinotecan / Leucovorin	0 (0.0%)	0 (0.0%)	1 (0.9%)	1 (0.5%)
5-FU / Gamma Interferon / Leucovorin	2 (5.3%)	0 (0.0%)	0 (0.0%)	2 (1.1%)
5-FU / Interferon / Leucovorin	2 (5.3%)	0 (0.0%)	0 (0.0%)	2 (1.1%)
5-FU / Irinotecan / Leucovorin	6 (15.8%)	13 (34.2%)	29 (27.4%)	48 (26.4%)
5-FU / Irinotecan / Leucovorin / Oxaliplatin	0 (0.0%)	0 (0.0%)	5 (4.7%)	5 (2.7%)
5-FU / Irinotecan / Leucovorin / Oxaliplatin / Capecitabine	0 (0.0%)	0 (0.0%)	2 (1.9%)	2 (1.1%)
5-FU / Irinotecan / Leucovorin / Capecitabine	2 (5.3%)	0 (0.0%)	4 (3.8%)	6 (3.3%)
5-FU / Leucovorin	2 (5.3%)	1 (2.6%)	3 (2.8%)	6 (3.3%)
5-FU / Leucovorin / Oxaliplatin	3 (7.9%)	3 (7.9%)	38 (35.8%)	44 (24.2%)
5-FU / Leucovorin / Oxaliplatin / Capecitabine	0 (0.0%)	0 (0.0%)	1 (0.9%)	1 (0.5%)
Bevacizumab Only	0 (0.0%)	0 (0.0%)	2 (1.9%)	2 (1.1%)
Cetuximab Only	0 (0.0%)	4 (10.5%)	0 (0.0%)	4 (2.2%)
Floxuridine / Irinotecan	1 (2.6%)	0 (0.0%)	0 (0.0%)	1 (0.5%)
Irinotecan	11 (28.9%)	13 (34.2%)	3 (2.8%)	27 (14.8%)
Irinotecan / Oxaliplatin / Capecitabine	0 (0.0%)	0 (0.0%)	1 (0.9%)	1 (0.5%)
Irinotecan / Capecitabine	0 (0.0%)	2 (5.3%)	3 (2.8%)	5 (2.7%)
Oxaliplatin / Capecitabine	2 (5.3%)	1 (2.6%)	7 (6.6%)	10 (5.5%)
Capecitabine	6 (15.8%)	1 (2.6%)	6 (5.7%)	13 (7.1%)

### Patient care monitor assessment

There were 1236 valid PCM assessments available from the 182 patients in the study. Baseline scores were available for 90.6% of patients (n = 165), who provided a total of 1156 PCM assessments for analysis that controlled for baseline symptom scores.

### Moderate to severe symptomatology

Many patients experienced moderate to severe symptoms, defined by PCM item scores ≥ 4, at some point during second-line therapy. Fatigue was the most common, occurring at moderate to severe levels in 67% of patients. The Cetuximab group reported a significantly higher rate of moderate to severe dry skin (p < .0001), itching (p = .0028), and rash (p < .0001), with rash rates of 60.5% in the Cetuximab group vs. 7.5% in the Bevacizumab group. Patients on Chemotherapy Only had a higher rate of moderate to severe nausea (p = .0485) than the Bevacizumab group, and tended to have a higher rate of physical pain (p = .0564) than the Bevacizumab group. The Cetuximab group tended to have a lower rate of moderate to severe problems with sweating than did the Bevacizumab group (p = .0637). Groups did not differ significantly on other symptoms.

### Linear mixed models analysis of PCM items

#### Baseline analyses

Linear mixed models were run twice, with one set of models controlling for baseline scores and a second set of models not controlling for baseline scores. Treatment group was significant in almost all of the models that did not control for baseline scores. However, after inclusion of baseline scores, those results changed notably as baseline symptom burden scores were the strongest predictor of second-line symptom burden across all measured items (all p’s < .0001 except for Rash, p = .0013).

#### Rash

**Figure 2:**
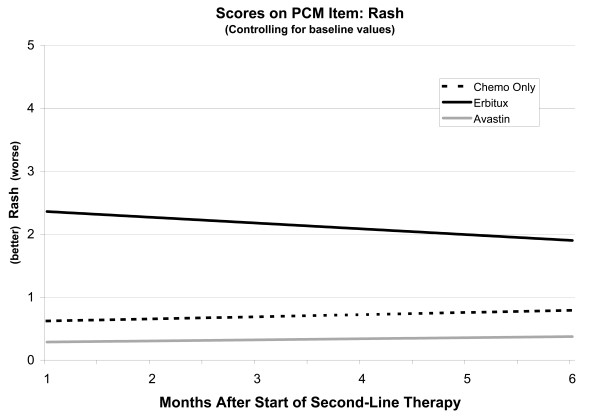
Linear mixed model of rash scores controlling for baseline.

As evidenced in Figure 2, patients in the Bevacizumab group had significantly (p < .0001) lower (better) Rash scores than did patients in the Cetuximab group and nominally lower scores than patients in the Chemotherapy Only group. Rash scores in the Cetuximab group fell faster than those in the Bevacizumab group (p = .019), albeit from a higher level. It should be noted that scores in the Bevacizumab group were near enough to zero that they had little room to fall. Although baseline rash scores significantly predicted rash scores during second-line therapy (p = .0013), membership in the Cetuximab group was a very strong predictor. Longer duration of first-line therapy was associated with less severe rash symptoms (p = .0375). Patients on either irinotecan or oxaliplatin had less severe symptoms (p = .0001, p = .0005, respectively) than patients on neither agent.

#### Dry skin

**Figure 3:**
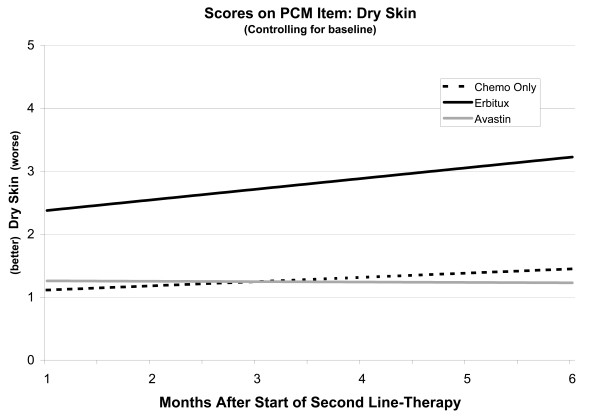
Linear mixed model of dry skin scores controlling for baseline.

There was a significant effect of Group (p < .0013), Interval (p = .01), and the interaction of the two (p = .0128) on Dry Skin scores (Figure 3), controlling for baseline. This suggests that Bevacizumab and Chemotherapy Only patients had low and stable scores whereas Cetuximab patients had Dry Skin scores that were significantly higher than the Bevacizumab group (p = .0008), and that also were changing (worsening) at a rate significantly different from the Bevacizumab group (p = .0037). Baseline scores were also a significant predictor in this model. Baseline adjusted scores for the Chemotherapy Only group were comparable to those of the Bevacizumab group whereas, when not controlled, they appeared significantly elevated. The implication of this result is that Chemotherapy Only patients may be more likely than Bevacizumab patients to enter therapy with elevated Dry Skin ratings. For those who do, elevated scores may persist. However, it is the elevated baseline scores that drive elevated scores during second-line therapy, not their group membership. In contrast, Cetuximab patients had higher scores than patients receiving bevacizumab, regardless of whether or not we controlled for baseline values.

#### Itching

**Figure 4:**
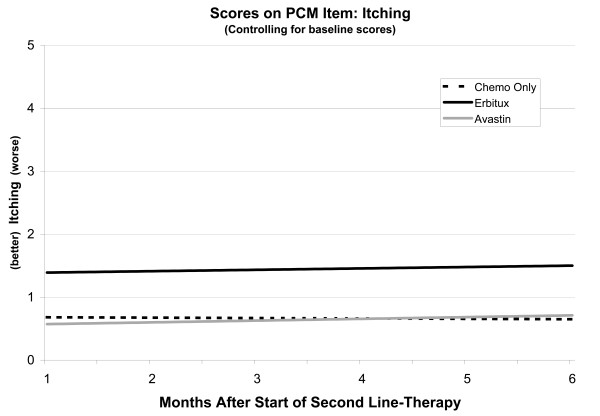
Linear mixed model of itching scores controlling for baseline.

Itching scores tended to be stable across treatment groups (Figure 4). Baseline scores were a significant predictor in this model (p < .0001). Controlling for baseline scores, the Bevacizumab group scored significantly lower (better) than the Cetuximab group (p = .0002), and no different from the Chemotherapy Only group. As in the model for Dry Skin, baseline adjusted scores for the Chemotherapy Only group were comparable to those of the Bevacizumab group, but scores not adjusted for baseline were elevated compared to the Bevacizumab group. As with the analysis of Dry Skin, the implication is that the baseline scores of Chemotherapy Only patients, and not their group membership, dictated scores during second-line therapy. In contrast, for the Cetuximab patients, their membership in the Cetuximab group predicted higher scores during second-line chemotherapy, independent of their baseline scores.

#### Nail changes

There was a significant main effect of Interval (p = .0292), and a near significant interaction of Group with Interval (p = .0587) suggesting that the Bevacizumab and Chemotherapy Only groups basically had stable Nail Change scores that did not significantly differ, controlling for baseline. Conversely the Cetuximab group had scores that were increasing, representing a rate of change over time that was significantly greater than that of the Bevacizumab group (p = .0182). Baseline scores positively predicted scores during treatment (p < .0001).

#### Nausea

There was a significant but modest main effect of Interval (p = .0188), in which Nausea scores improved slightly over time. The effect represented less than 1 point improvement over a one year period with little notable difference among groups. Baseline scores did significantly predict Nausea scores over time during second-line treatment (p < .0001). There also was a small effect associated with duration of first-line treatment (p = .022), in which longer first-line treatment (e.g. 2 months) was associated with somewhat milder (0.1 point improvement) nausea symptoms during second-line therapy.

#### Vomiting

There was a significant but slight main effect of Interval (p = .0007), with Vomiting scores decreasing (improving) over time. However, the effect was very small (e.g. a 0.1 point improvement over a 5 month period, well short of a clinically significant effect), with very mild symptoms overall. There was no significance in the main effect of Group, or in the interaction of Group and Interval. Baseline scores strongly predicted Vomiting scores during treatment (p < .0001).

#### Diarrhea

There was a significant effect of Interval (p < .0001), controlling for baseline, with scores improving over time. There was no significant main effect of Group, and no significant interaction between Interval and Group.

#### Burning sensation in hands & feet

There were no significant main effects or interaction between Group and Interval on Scores for Burning Sensation in Hands & Feet. Baseline scores did predict scores during second-line but with or without control of baseline, scores were relatively low, indicating mild symptoms, and relatively stable scores.

#### PCM index scores

Models assessing group differences on PCM index scores were also examined. In general, due to the composite nature of these measures, and the specificity of effects described in the foregoing section, any group differences were washed out on these endpoints. As noted earlier, the individual PCM items were the primary focus of the investigation.

## Discussion

This study examined retrospectively collected medical record data, and repeated symptom burden assessments, from CRC patients with metastatic disease treated in the second-line setting with regimens containing Bevacizumab, Cetuximab, or Chemotherapy Only at seven community oncology practices in the United States (U.S.).

The results suggest that baseline symptom burden scores are the strongest single predictor of second-line symptom burden across the items measured in this study. However, after controlling for baseline symptom scores, the data suggest that patients on a Bevacizumab containing regimen tended to have more stable and less severe symptoms as measured by PCM items than patients on a Cetuximab containing regimen, and than patients on Chemotherapy Only in several areas.

This study adds to the literature in several ways. By assessing key symptoms with repeated assessments, this study was able to characterize symptom burden specifically during second-line therapy, after patients had received prior treatment and experienced disease progression. The study was able to directly compare symptom burden in key areas across regimens that included two widely used biologics, and across regimens that included chemotherapy only without monoclonal antibodies. The study was also able to show that some symptoms present at initiation of second-line therapy persist, and that symptoms assumed to reflect treatment group effects may be attributable to group differences in symptom burden at initiation of second-line therapy. The study was unable to identify obvious demographic or clinical differences between patients in the treatment groups. Given that there were group differences in self-reported symptom burden at start of second-line therapy, this suggests that these differences may not be easily identifiable other than by self-report, or that they arose during first-line therapy. Clinicians should be aware of both possibilities.

Elevated dermatological symptoms during second-line therapy were observed in both the Cetuximab and Chemotherapy Only groups, but were substantially explained by baseline scores for the Chemotherapy Only group. This was not true of the Cetuximab group, however. The data do not reveal why patients with elevated scores at baseline had these elevated scores—there were no identifiable differences across treatment groups in first-line regimen, or in the duration of first-line therapy. Regardless, the data do suggest that patients in the Chemotherapy Only group who did not have elevated rash and dry skin symptoms at baseline tended not to report such problems during second-line therapy, and that this claim is not supported for the Cetuximab group.

Although PCM index scores may be less sensitive to specific effects due to their composite nature, previous research has shown them to be sensitive endpoints in other cancer populations [26,30,31]. As noted, these were not the focus of the analysis, but given findings in prior research, the absence of findings for PCM index scores was somewhat unexpected.

In interpreting these findings, the following factors should be considered. First, although data were collected from a number of geographically dispersed oncology clinics in the U.S., the study used a convenience sample that may differ in unknown ways from the underlying population. Also, the observational nature of this research involved comparison of existing groups, with the possibility that there were pre-existing group differences related to the study outcomes. For example, it is possible that patients treated with Bevacizumab may have had some unknown selection factor operating such that patients with fewer pre-existing risk factors for skin related toxicities were also more likely to receive Bevacizumab than either Cetuximab or Chemotherapy Only. In this and other ways, selection bias may therefore have affected the direction and magnitude of group differences observed. A further limitation is that the comparison groups were unequal in sample size with the Bevacizumab group having 106 cases compared to the Cetuximab and Chemotherapy only groups with 38 cases each. This reduced the statistical power of comparisons among groups relative to a balanced study design. Finally, we examined patients with mCRC treated in community practice settings. Results may therefore not generalize to patients with other diseases, with disease at other stages, and to patients treated in other settings.

## Conclusions

The biggest predictor of second-line symptom burden is the symptom burden observed at the end of the first-line regimen. Patients receiving a second-line therapy for mCRC which contained bevacizumab reported fewer treatment-related symptoms as compared to patients receiving a regimen containing cetuximab.

## Clinical practice points

Treatment side effects common to systemic cancer therapy may be present at the start of second-line treatment of mCRC. The severity of these symptoms is predictive of the severity of symptoms patients will self-report as present during the course of second-line therapy. Although there are differences in the pattern of symptoms patients may experience across regimens and with different biologic therapies, differences in side effect profile across regimens during second-line mCRC may reflect baseline differences in symptom burden. These symptoms may be attributable to the first-line regimens received, and may not be evident other than through patient self-report. Additional discussion of symptom severity with patients may be useful in this regard.

## Competing interest

The study reported in this paper was funded by Genentech, Inc. Elaine Yu, Pharm.D. and Yeun Mi Yim, M.P.H. are employed by and own stock in Genentech. Lee S. Schwartzberg, M.D. is a consultant for Roche. Genentech is a member of the Roche Group. The other authors report no conflicts of interest.

## Authors' contributions

MW provided overall research design and scientific supervision. EY assisted with research design and scientific background. JK provided statistical analysis and quality control. YY provided background research and assisted with drafting the manuscript. ES provided research design consultation and measurement review. LS provided clinical background and assisted with research design and measurement decisions. All authors read and approved the final manuscript.
